# Singular v Dual inhibition of SNF2L and its isoform, SNF2LT, have similar effects on DNA Damage but opposite effects on the DNA Damage Response, Cancer Cell Growth Arrest and Apoptosis

**DOI:** 10.18632/oncotarget.479

**Published:** 2012-05-09

**Authors:** Yin Ye, Yi Xiao, Wenting Wang, Jian-Xin Gao, Kurtis Yearsley, Quintao Yan, Sanford H. Barsky

**Affiliations:** ^1^ Department of Pathology, University of Nevada School of Medicine, Reno, NV; ^2^ Department of Pathology, Ohio State University College of Medicine, Columbus, OH; ^3^ The Whittemore-Peterson Institute, Reno, NV; ^4^ Nevada Cancer Institute, Las Vegas, NV

**Keywords:** SNF2L, DNA damage, SNF2LT, NURF

## Abstract

SNF2L, an ATPase chromatin remodeling gene nearly ubiquitously expressed in diverse tissues, cancers, and derived cell lines, contributes to the chromatin remodeling complex that facilitates transcription. Because of this near ubiquitous expression, it has not been exploited as a cancer therapeutic target. However, in a recent study, we found that highly malignant cancer cells, although expressing SNF2L at similar levels as their normal counterparts, were sensitive to its knockdown. Only the highly malignant (HM) lines showed significant growth inhibition, DNA damage, a DNA damage response, and phosphorylation of checkpoint proteins and marked apoptosis. In studying SNF2L, we discovered a novel truncated isoform, SNF2LT which, when compared to full length SNF2L, lacked three important domains: HAND, SANT and SLIDE. Although truncated isoforms usually have antagonistic functions to their parental molecule, here SNF2LT knockdown had similar effects to the knockdown of its parental molecule, SNF2L, of inducing DNA damage, a DNA damage response, cell cycle arrest and apoptosis selectively in cancer cell lines. However dual SNF2L and SNF2LT knockdown, while inducing DNA damage, did not result in a DNA damage response, a cell cycle arrest and apoptosis. In fact HM lines subjected to dual knockdown paradoxically exhibited sustained cell growth. Our findings indicate that the ratio of SNF2L to its isoform tightly regulates the cancer cell's response to DNA damage. Cancer cell lines which endogenously express low levels of both SNF2L and its isoform mimic the situation of dual knockdown and permit DNA damage which is allowed to propagate unchecked.

## INTRODUCTION

Aberrant gene function and altered patterns of gene expression are key features of cancer. An explosion of data indicating the importance of epigenetic processes, especially those resulting in the silencing of key regulatory genes, has led to the realization that genetics and epigenetics cooperate at all stages of cancer development [1-5]. Epigenetic modifications fall into two main categories: DNA methylation and histone modifications [6-10]. The processes of covalent histone modification and DNA methylation couple with chromatin remodeling by ATP-dependent remodeling machines to obtain efficient transcriptional regulation, DNA replication and DNA-damage repair [11-15]. Emerging data have shown that ATPase-dependent remodeling enzymes all act in the context of multisubunit complexes, which adds an additional layer of fine-tuned specificity in ATP-dependent chromatin remodeling [16-20].

SNF2L (SMARCA1), an ATPase chromatin remodeling gene nearly ubiquitously expressed in diverse tissues, cancers, and derived cell lines, contributes to the chromatin remodeling complex that facilitates transcription. Because of this near ubiquitous expression, it has not been exploited as a cancer therapeutic target. However, in a recent study [21], we found that cancer cells, although expressing SNF2L at similar levels as their normal counterparts, were exquisitely sensitive to its knockdown. This was not observed when its imitation SWI ortholog, SNF2H, was inhibited. SNF2L siRNA inhibition using two different siRNAs separately reduced SNF2L transcript levels and protein in both normal and cancer lines, but only the cancer lines showed DNA damage, a DNA damage response, phosphorylation of cell cycle checkpoint proteins, significant growth inhibition and marked apoptosis.

SNF2L, the ISWI family member of the SNF2 ATPase superfamily in humans, is one subunit of the ATP-dependent chromatin complex hNURF. The other two subunits of hNURF are BPTF and RbAP46/RbAP48 [22]. The data have shown that NURF regulates expression of homeotic genes, modulates Wnt-signaling, and affects higher-order chromatin structure; *in vitro* NURF catalyzes formation of regularly spaced nucleosomal arrays and facilitates transcription activation [23,24].

The SNF2L gene encodes a 1054-amino acid protein with six putative conserved domains: SNF2_L, DEXHc, HELICc, HAND, SANT and SLIDE [25]. The SNF2_N (SNF2 family N-terminal domain) (186-466 aa) is found in proteins involved in a variety of processes including transcription regulation, DNA repair, DNA recombination, and chromatin unwinding. DEXHc contains the ATP-binding region and is found in a diverse family of proteins involved in ATP-dependent DNA or RNA unwinding. HELICc (Helicase superfamily c-terminal domain) (477-620 aa) is found in a wide variety of helicases and helicase related proteins; it utilizes the free energy from nucleoside triphosphate hydrolysis to fuel their translocation along DNA, unwinding the duplex in the process. HAND (the HAND domain) (758-838 aa) adopts a secondary structure consisting of four alpha helices, three of which (H2, H3, H4) form an L-like configuration. The domain confers DNA and nucleosome binding properties to the protein. Tandem copies of the SANT (‘SW13, ADA2, N-CoR and TFIIIB’ DNA-binding domains) domain bind telomeric DNA tandem repeats as part of the capping complex. Binding is sequence dependent for repeats which contain the G/C rich motif [C_2-3_A(CA)_1-6_]. The domain is also found in regulatory transcriptional repressor complexes where it also binds DNA. The SLIDE domain (913-1022 aa) adopts a secondary structure comprising a main core of three alpha-helices. It has a role in DNA binding, contacting DNA target sites similar to c-Myb repeats or homeodomains. SANT and SLIDE domains also mediate interaction with unmodified histone tails and linker DNA.

In studying SNF2L, we discovered a novel truncated isoform, SNF2LT, which, when compared to full length SNF2L lacked three important domains: HAND, SANT and SLIDE. Because SNF2L knockdown had such a selective effect on highly malignant (HM) lines [21) and the presence of an altered functional splice variant might suggest an additional level of biological complexity in the regulation of the action of SNF2L, we decided to study both molecules and their relationship in detail in the present study.

## MATERIALS AND METHODS

### Reagents and antibodies

All antibodies used were rabbit polyclonal unless otherwise indicated and included an antibody to SNF2L (SMARCA1; ab37003; Abcam, Inc.). Antibodies used for detection of DNA damage were obtained from the DNA Damage Antibody Sampler (Cell Signaling Technology, Inc., Danvers, MA), which included Phospho-ATR (Ser428), Phospho-ATM (Ser1981) mouse monoclonal antibody (mAb), Phospho-BRCA1 (Ser1524), Phospho-CHK1 (Ser296), Phospho-CHK2 (Thr68), Phospho-Histone H2AX (Ser139), and Phospho-p53 (Ser15; 16G8) mouse mAb. p53 (7F5) rabbit mAb and β-actin (13E5) rabbit mAb were also used (Cell Signaling Technology, Inc.). Antibodies for detection of cell cycle checkpoints used the Cell Cycle/Checkpoint Sampler kit (Cell Signaling Technology, Inc.), which included Phospho-cdc2 (Tyr15) and Phospho-Rb (Ser795). Additional antibodies that recognized total protein levels of the respective DNA damage proteins (CHK1, CHK2, BRCA1, ATR, and H2AX) and cell cycle checkpoint proteins (cdc2, Rb, mouse mAb) were also obtained (Cell Signaling Technology, Inc.). Secondary antibodies and Western blotting substrates were obtained (Pierce Biotechnology, Inc., Rockford, Il). Human tumor total RNAs and the FirstChoice Human Total RNA Survey Panel were also used (Ambion, Inc., Austin, TX).

### Cell lines and human tissues

All the cell lines were grown under standard conditions in DMEM with 10% fetal bovine serum with the exception of the fibroblast lines, which were grown in MEM-α medium with 10% fetal bovine serum, and the myoepithelial lines, which were grown in KSFM with supplements (Life Technologies, Inc., Grand Island, NY). All lines, unless otherwise indicated, were obtained from a single source (American Type Culture Collection, Manassas, VA) and were human and consisted of the following: HM: estrogen receptor–negative breast cancer lines (MDA-MB-231, MDA-MB-468), Her-2/neu–amplified breast cancer lines (HCC202, HTB20; HTB27), an inflammatory breast cancer xenograft, MARY-X, established by us [26,27], a cervical squamous cell carcinoma (HeLa), a rhabdomyosarcoma (RB), a leiomyosarcoma (SKLMS-1), and an osteosarcoma (U2-0S) line; NU: three fibroblast lines including dermal HDF and pulmonary HLF, (gifts of Dr. Issekutz, Dalhousie University, Halifax, Canada) and skin OSU-2 (a gift of Altaf Wani, Ohio State University, Columbus, OH), a mammary epithelial line (HMEC; Clonetics), and the nontumorigenic estrogen receptor–negative MCF-10A line; and benign or LG lines and xenografts: HMS-X and HMS-1, derived by us from a benign human myoepithelial salivary gland tumor and HMS-3X, HMS-4X, and HMS-6X, derived from other benign matrix-secreting human myoepithelial tumors from salivary gland, breast and pulmonary sources [28], and the estrogen receptor–positive breast carcinoma MCF-7. All the lines were grown under standard conditions.

Human tissues (normal and tumoral) were obtained from the frozen tissue bank of the Human Tissue Network at the Ohio State University.

### siRNA transfections

The Silencer Pre-designed siRNA against human SNF2L (ID#12578) was obtained (Ambion, Inc.). The target site of siRNA was Exon 2 of SNF2L. The sequences of siRNA oligonucleotide duplex were as follows: 5’-GGAAAUGGACCCAGAAUAUTT-3’ (sense) and 5’-AUAUUCUGGGUCCAUUUCCTT-3’ (antisense). The siRNA oligonucleotide duplex targeted to SNF2LT (A1IS) was synthesized (Integrated DNA Technologies, Inc., Coralville, IA) and their sequences were as follows: 5’-CAUGAUCUAUGGGUCAGAUUU-3’ (sense) and 5’-AUCUGACCCAUAG AUCAUGUU-3’ (antisense). The target site of siRNA (ID#12667) was exon 18 of SNF2LT but exon 19 of SNF2L. Negative control siRNA (ID#AM4611) (NCSI) was obtained (Ambion, Inc.). Cells were reverse transfected with siRNA (50 nM) using Lipofectamine RNAiMAX Transfection Reagent (Invitrogen Corporation, Inc.).

### Plasmid constructions

Human full-length SNF2L ORF cDNA was synthesized by RT-PCR using the human breast carcinoma cell line MDA-MB-468 cDNA as template. SNF2L cDNA and SNF2LT were separately cloned into vector pCR2.1-TOPO (Invitrogen, Inc., Carlsbad, CA) and sequenced. The SNF2LT ORF was subcloned into pcDNA™6.2/Myc-His-A to construct the SNF2LT expression vector pcDNA™6.2/SNF2LT-Myc-His with the C-terminal myc epitopes and the polyhistidine tags. This vector was transfected directly into cultured cells using Lipofectamine 2000 (Invitrogen, Inc.). (See Supplementary Information on line).

### Cell growth, cell cycle and apoptosis experiments

Cells were transfected with the different siRNAs and seeded in 24-well cell culture plates. The number of viable cells in each well was counted every 24 h for 3 d using trypan blue exclusion. The cell growth study was carried out in triplicate and repeated at least four times. For cell cycle analysis, the cells were collected 12 to 24 h after transfection and fixed in 70% ethanol at −20°C, followed by washing once in PBS and staining in PI solution (69 mmol/L PI, 388 nmol/L sodium citrate, 100 μg/mL RNase A) for 15 min at room temperature. Ten thousand cells were analyzed on Coulter Epics XL flow cytometer (Beckman Coulter, Inc., Brea, CA). For the apoptosis assay, cells were harvested at 48 to 72 h following transfection. The apoptosis assay used Annexin V-FITC and PI (kit PN IM2375, Beckman Coulter, Inc.) with flow cytometric analysis.

### DNA damage and the DNA damage response with apoptosis inhibition

To determine the order of cellular events with SNF2L, SNF2LT or dual knockdown, selected cell lines, e.g., MDA-MB-468 cells, were seeded in six-well plates and incubated in 37°C overnight. Cells were treated first with general caspase inhibitors (Caspase Inhibitor Set IV, EMD Chemicals, Billerica, MA) for 45 min and then with the different siRNA’s for 24 h. Treated cells were collected and divided into three aliquots: the first aliquot was analyzed for apoptosis; the second aliquot was studied for DNA damage by the CometAssay; and a third aliquot was analyzed for protein levels of p-H2XA.

### Alkaline comet assay

The CometAssay (single-cell gel electrophoresis assay; Trevigen, Inc., Gaithersburg, MD) was used to evaluate DNA damage. The technique used electrophoresis of lysed cells embedded in an agarose gel, diluted in a SYBR green solution and viewed by DNA fluorescence. Cells with damaged DNA exhibited migration of their DNA outside of the nucleus, producing a comet tail.

### RNA isolation and cDNA synthesis

The total RNA was isolated from cultured cells using RNeasy Mini Kit (Qiagen Inc., Valencia, CA). Total RNA was dissolved in RNase-free water and the concentration determined by measuring absorbance using Nanodrop spectrophotometer at 260 nm. For first strand cDNA synthesis, SuperScript® III First-Strand Synthesis System (Invitrogen, Inc.), ologo (dT)20 and 1 μg of total RNA were used. The synthesized cDNA was used for regular RT-PCR or real-time PCR analysis of relative expression levels of target genes.

### RT and real-time PCR

An aliquot of 20 ng cDNA was used in each 25 μL PCR reaction, using Platinum Taq DNA Polymerase High fidelity (Invitrogen, Inc.). The following conditions used were as follows: denaturation at 94°C for 30 s, annealing at 58°C for 30 s, and extension at 68°C for 1 min for a total of 25, 30, or 35 cycles. PCR products were analyzed by 2.0% agarose gel. Real-time PCR was done on a ABI 7500 Real-time PCR System (Applied Biosystems, Inc., Foster City, CA). cDNA was combined with primer sets and Power SYBR Green PCR Master Mix (Applied Biosystems, Inc.) was used. Gene expression levels were calculated relative to the housekeeping gene β-actin (ACTB) by using 7500 System SDS software (Applied Biosystems, Inc.). Primer sets (forward and reverse) used for either RT-PCR or real-time PCR included the following (forward, reverse):

**Human SNF2L**
5’-ACGGCCTCCAAAACAGCCAAATG-3’,
5’-TGAGCCAGAGCTGGATTTGGGATA-3’

**ATM**
5’-TGGATCCAGCTATTTGGTTTGA-3’,
5’-CCAAGTATGTAACCAACAATAGAAGAAGTAG-3’

**ATR**
5’-TGTCTGTACTCTTCACGGCATGTT-3’,
5’-AGAGGTCCACATGTCCGTGTT-3’

**CHK1**
5’-GGTGAATATAGTGCTGCTATGTTGACA-3’,
5’-TTGGATAAACAGGGAAGTGAACAC-3’

**CHK2**
5’-AGTGAGAGGACTGGCTGGAGTT-3’,
5’-CCCAAGGCTCCTCCTCACA-3’

**TP53**
5’-TCAACAAGATGTTTTGCCAACTG-3’,
5’-ATGTGCTGTGACTGCTTGTAGATG-3’

**14-3-3σ**
5’-TGCTGCCTCTGATCGTAGGAATTG-3’,
5’-TTCCCTCAATCTCGGTCTTGCACT-3’

**GADD45A**
5’-TCAGCGCACGATCACTGTC-3’,
5’-CCAGCAGGCACAACACCAC-3’

**APAF-1**
5’-GCATCACCCTTTGTAATAAC-3’,
5’-CCCAGCTAATTTTTGTAGTT-3’

**BAD**
5’-TTAAACCTGGCTCGCGACTT- 3 ‘,
5’-GTGCTGTCTCCTTTGGAGGG-3’;

**BAX**
5’-CCTTTTCTACTTTGCCAGCAAAC-3’,
5’-GAGGCCGTCCCAACCAC-3’

**BIK**
5’-CTTGATGGAGACCCTCCTGTATG-3’,
5’-AGGGTCCAGGTCCTCTTCAGA-3’

**BAK1**
5’-GAACAGGAGGCTGAAGGGGT-3’,
5’-TCAGGCCATGCTGGTAGACG-3’

**BID**
5’-GGTCTTACAGCAGGCAGTATCC-3’,
5’-TCAGAATCTCTGTGCCATGTG-3’

**BCL2**
5’-GGAACAATGCAGCAGCCGAG-3’,
5’-GTAGAGTGGATGGTCAGTGT-3’

**CASP1**
5’AATACTGTCAAATTCTTCATTGCAGATAA-3’,
5’-AAGTCGGCAGAGATTTATCCAATAA-3’

**CASP3**
5’-AGAACTGGACTGTGGCATTGAG-3’,
5’-GCTTGTCGGCATACTGTTTCAG-3’

**CASP6**
5’-ACCTCCCACACTGGGAACCACA-3’,
5’-CACCTGTATGACCAATTCCATGTC-3’

**CASP7**
5’-AGTGACAGGTATGGGCGTTCG-3’,
5’-GCATCTATCCCCCCTAAAGTGG-3’

**CASP8**
5’-CCTGGGTGCGTCCACTTT-3’,
5’-CAAGGTTCAAGTGACCAACTCAAG- 3’

**CASP9**
5’-ATAACCTTTTAGGCTGGTGG-3’,
5’-AGAGCAGAAAGAGGTGAGAGA-3’

**CASP10**
5’-CCCTTAAACATTGGACAGTG-3’,
5’-GTGTAAATGAGCCATCATCTTC-3’

**ACTB**
5’-GGCACCCAGCACAATGAAG-3’,
5’-GCCGATCCACACGGAGTACT-3’

### Preparation of protein lysates and western blot analysis

To prepare protein lysates from cultured cell lines for western blot analysis, cells were lysed using ice-cold RIPA lysis buffer (50 mM Tris, 150 mM NaCl, 50 mM NaF, 1 mM Na4P2O7•10 H2O, 0.1% DOC, 1.0% NP-40, 50 μl Na3VO4, and Halt Protease Inhibitor Cocktail) (Pierce Biotechnology, Inc, Rockford, IL)). After 15 min on ice with shaking, the lysates were centrifuged at 15000 × g for 10 min at 4°C. Supernatants were stored at -80°C until use. For western blot analysis, protein concentrations were determined using the BCA Protein Assay (Pierce Biotechnology, Inc). Equal amounts of boiled protein were loaded onto a 4-12% Precast gradient gel (Invitrogen, Inc.) and transferred to nitrocellular membranes. The membranes were washed in TBST buffer. Membranes were then washed and incubated with secondary antibody for 1 hour at room temperature. Bound antibodies were detected by a chemiluminescent detection system (West Femto; Pierce Biotechnology, Inc.).

### Other studies

Additional studies of constitutive and conditional gene expression of SNF2L and SNF2LT were carried out with transient and stable transfection methods. (See Supplementary Information on line).

### Institutional approvals and human tissues

Use of human tissues was approved by The Ohio State University Cancer Institutional Review Board under protocol 2006C0042. Specifically, select normal and cancerous tissues were obtained from an anonymized frozen tissue bank. All animal and *in vitro* studies were approved by The Ohio State University’s Animal Care and Use Committee (Institutional Animal Care and Use Committee), protocol 2007A0218, and by the Institutional Biosafety Committee, protocol 2007R0057. Additional animal studies were approved by the University of Nevada, Reno’s Institutional Animal Care and Use Committee, protocols 00439 and 00440.

### Statistical analysis

All experiments performed were subjected to statistical analyses. Each experiment was performed in triplicate and repeated at least four times. Representative results were depicted. Declarations of differences imply differences of statistical significance. Significance was assessed by the Student’s t-test.

## RESULTS

### Serendipitous discovery of human SNF2LT (A1lS), a novel truncated isoform of human SNF2L

**Figure 1:**
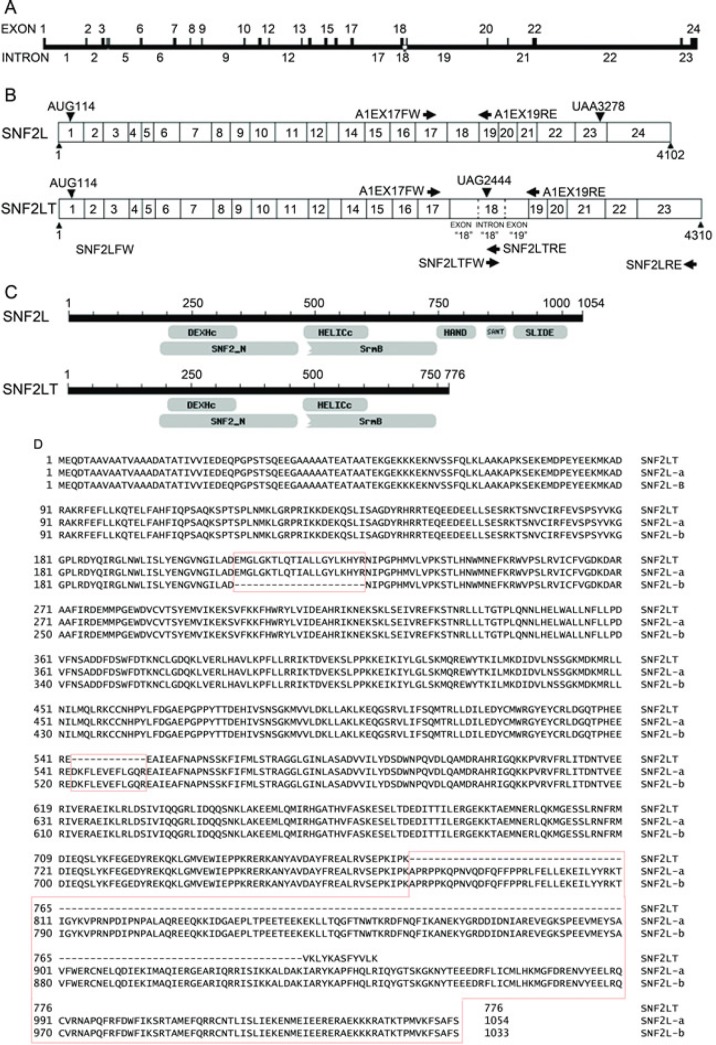
Comparisons of SNF2L (SMARCA1) with SNF2LT (A1IS) A, the exon/intron map of SNF2L/SNF2LT is depicted. B, the RT-PCR strategy to amplify both SNF2L and SNF2LT is depicted. C, the structural/functional domains of SNF2L are compared to those of SNF2LT. D, detailed comparisons of intron and exon sequences present in SNF2L (SMARCA1) and SNF2LT (A1IS). Two variants of parental SNF2L (SNF2L-a and SNF2L-b) are depicted. Each experiment was performed in triplicate and repeated at least four times.

In initially studying SNF2L expression, it was noted that the expression levels of SNF2L were highest in ovary and testis [21]. The exon-intron map of SNF2L is depicted (Figure 1A). RNA from ovary was subjected to RT-PCR with primers which annealed to exon 17 located at 2097-2120 nt of SNF2L (primer A1EX17FW) and exon 19 at 2367-2384 nt (primer A1EX19RE) (Figure 1B). The amplified products were separated on a 1.5% agarose gel and stained with ethidium bromide (data not shown). As expected, one amplification product of approximately 300 bp was observed as the SNF2L cDNA product. Interestingly, we found another amplification product about 550 bp in length. This 550-bp product was excised from the gel and purified and sequenced. Nucleotide sequencing revealed that the 5’end and the 3’end of the 550-bp product were the sequences of SNF2L cDNA. However, the central 250 bp fragment of this product did not match the sequence of SNF2L cDNA. We performed a blast analysis using the 250 bp fragment sequence on the NCBI website and discovered that this 250 bp fragment was the whole sequence of intron 18 of the SNF2L gene. This raised the possibility that we might have stumbled on a novel alternatively spliced isoform of SNF2L. To further confirm the new alternatively spliced mRNA containing intron 18 of SNF2L, we used two pairs of primers for RT-PCR that covered intron 18 (Figure 1B). The primer SNF2LFW (A1FW) which annealed to the 5’end of SNF2L cDNA and SNF2LTRE (A1ISRE) which annealed to the 3’end of intron 18 were used to amplify the sequence that covered the 5’end of SNF2L and a significant portion of intron 18. The primer SNF2LTFW(A1ISFW) which annealed to the 5’end of intron 18 and SNF2LRE(A1RE) which annealed to the 3’end of SNF2L cDNA were used to amplify the sequence that covered the 3’end of SNF2L and a significant portion of intron 18. The amplified products were again separated on a 1.5% agarose gel and stained with ethidium bromide. As expected, two amplification products were observed: one approximately 2500-bp from primers SNF2LFW(A1FW) and SNF2LTRE(A1ISRE) and one about 1800-bp from primers SNF2LTFW(A1ISFW) and SNF2LRE(A1RE) (data not shown). The products were again excised from the gel and purified and sequenced. The nucleotide sequence confirmed that the full-length cDNA was a novel alternatively spliced mRNA isoform (named as SNF2LT(A1IS)) that contained full length intron 18 (Figure 1B). Blast analysis of SNF2LT(A1IS) revealed that this isoform had not been previously reported. As schematically shown (Figure 1B), SNF2LT mRNA is 4310 nucleotides in length compared to 4102 nucleotides of SNF2L mRNA. Intron 18 of the SNF2L gene is not spliced out but exists in SNF2LT mRNA as an exon sequence. Because a stop codon is introduced in intron 18, SNF2LT encodes only a 776 amino acid protein that lacks the 262 amino acid C-terminal of SNF2L (Figure 1C; Figure 1D). SNF2LT also lacks 12 amino acids that correspond to amino acids 543–554 of the full length SNF2L protein (Figure 1D). Compared to the full length SNF2L protein, SNF2LT lacks three important domains: HAND, SANT and SLIDE (Figure 1C). We reported SNF2LT to GenBank (bankit1082498 EU36009).

### Nearly ubiquitous expression of SNF2LT in both normal and cancerous human tissues and their derived cell lines

**Figure 2:**
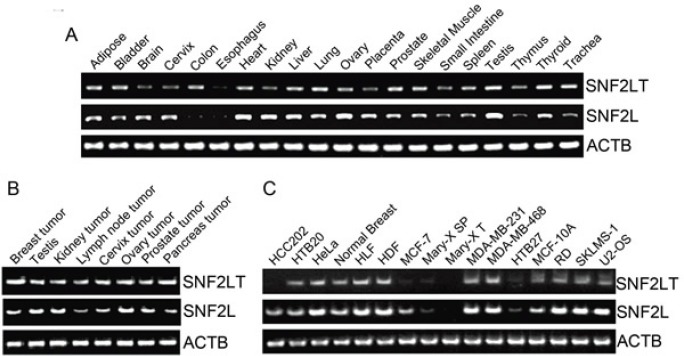
SNF2L and SNF2LT expression by RT-PCR in different normal human tissues, cancers and cell lines A, expression of SNF2L and SNF2LT by RT-PCR in different human normal tissues. SNF2L was nearly ubiquitously expressed in human normal tissues with especially high expression in ovary and testis; SNF2LT was similarly expressed though only at a molar fraction of the level of SNF2L. B, both SNF2L as well as SNF2LT were expressed in all tested human cancer tissues. C, both SNF2L and SNF2LT were also expressed in nearly all human cancer cell lines with the exception of MARY-XT (xenograft tumor) and *in vitro* derived spheroids (MARY-X SP), both derived from a patient with inflammatory breast cancer (26,27). ACTB served as housekeeping control. Each experiment was performed in triplicate and repeated at least four times.

Our previous results showed that human SNF2L was expressed nearly ubiquitously in both normal and human cancerous tissues and their derived cell lines [21]. Human SNF2LT was also expressed nearly ubiquitously in diverse normal (Figure 2A) and human cancerous tissues (Figure 2B) and their derived cell lines (Figure 2C) which included highly malignant (HM), low grade (LG) or benign and normal untransformed (NU) human cell lines. Compared to SNF2L mRNA, the level of SNF2LT mRNA was usually lower than the level of SNF2L, averaging about 50% -75% lower on a molar basis (Figure 2A; Figure 2B; Figure 2C). The expression of SNF2LT also paralleled the expression of SNF2L: when the expression of SNF2L was high, as it was in the majority of HM lines examined, the expression of SNF2LT was also high; when SNF2L expression was low, so was the expression of SNF2LT. Interestingly the expression of SNF2L as well as its SNF2L isoform were low to absent in MARY-X, a transplantable human xenograft derived from a case of inflammatory breast cancer [26]. In both MARY-X and its *in vitro* derived spheroids thought equivalent to *in vivo* lymphovascular emboli, the expression of both SNF2L as well as SNF2LT remained low to absent. Although SNF2L was detected in murine tissues in a prior study [21], using equivalent primers, murine SNF2LT could not be detected (data not shown). Therefore it appeared as if SNF2LT might be a human specific isoform.

### Singular v dual knockdowns of SNF2L/SNF2LT

**Figure 3:**
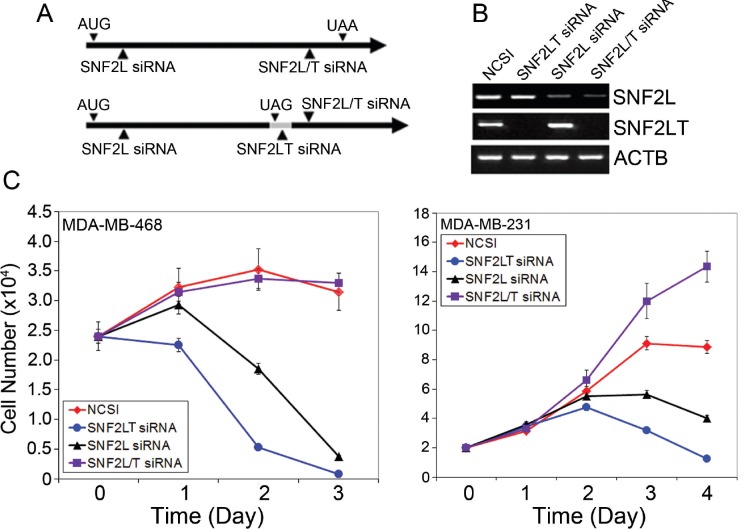
Singular v dual knockdown of SNF2L and SNF2LT and cell growth A, siRNAs and their targeted areas are depicted. B, SNF2L siRNA (12578) selectively knocked down SNF2L; SNF2LT siRNA (A1IS) selectively knocked down SNF2LT; and SNF2L/T siRNA (12667) knocked down both SNF2L as well as its isoform, SNF2LT. siRNA (NCSI) (RNAi-neg) was used as the negative control. ACTB served as housekeeping gene. C, singular knockdown of either SNF2L or SNF2LT significantly inhibited the growth of HM lines (MDA-MB-468 and MDA-MB-231). Dual knockdown of SNF2L as well as SNF2LT, however, was permissive of growth in HM lines. Each experiment was performed in triplicate and repeated at least four times.

Singular v dual SNF2L and SNF2LT knockdowns could be achieved with targeted siRNA oligonucleotide duplexes (Figure 3A): the Silencer Pre-designed siRNA against human SNF2L (ID#12578) targeted against Exon 2 of SNF2L; the siRNA oligonucleotide duplex targeted to SNF2LT (A1IS); and the siRNA (ID#12667) against exon 18 of SNF2LT and exon 19 of SNF2L. siRNA (NCSI) was used as a negative oligonucleotide duplex control. These specific oligonucleotide duplexes achieved specific singular knockdowns of either SNF2L (RNAi-12578) or SNF2LT (RNAi-A1IS) or dual knockdowns of both molecules (RNAi-12667) (Figure 3B). These results could be achieved in the diverse HM, LG or NU human cell lines which were examined. The specific knockdowns that were achieved were only partially predictable. A1IS siRNA targeted to “intron 18” that exists as an exon in A1IS mRNA but as an intron in SNF2L mRNA predictably knocked down SNF2LT (A1IS) but not SNF2L. 12578 siRNA targeted to the 5’ part of both SNF2L and A1IS mRNA and 12667 siRNA targeted to exon 18 of A1IS mRNA but exon 19 of SNF2L. Both 12578 and 12667 siRNAs might well be expected to separately knockdown both SNF2L as well as SNF2LT. However, 12578 siRNA selectively inhibited expression of SNF2L and had little effect on the expression of A1IS (Figure 3B). The molecular reasons for this selective knockdown were not clear. 12667 siRNA predictably led to the knockdowns of both SNF2L and A1IS (Figure 3B) and could be exploited to achieve dual knockdowns.

### Singular v dual knockdowns of SNF2L/SNF2LT and opposite effects on cell growth

After demonstrating that specific singular and dual knockdowns of full length SNF2L and its truncated isoform, SNF2LT could be achieved, we next examined the effects of these knockdowns on cell growth of a number of different cell lines including HM, LG and NU lines (complete list of lines examined provided in Materials and Methods). Our results showed that the growth of all the HM lines examined were dramatically inhibited when singular knockdowns of either SNF2L or SNF2LT were achieved (Figure 3C). In the HM lines, eg., MDA-MB-468 and MDA-MB-231, not only was there growth inhibition but the cell numbers were reduced by day 3 below starting numbers indicating that, in addition to the growth arrest, that induction of cell death or apoptosis had occurred. When one looks closely at the cell numbers, one finds that the growth of the cells subjected to SNF2LT knockdown was even more reduced than the growth of the same cells subjected to SNF2L knockdown (Figure 3C). In contrast, the LG and NU lines showed substantially less growth inhibition with no reduction in cell numbers. The growth rate of the HM lines were essentially the same when transfected with the negative control siRNA (Figure 3C). Dual knockdowns of SNF2L and SNF2LT, however, exhibited an increase in cell growth, findings dramatically opposite to the effects of singular knockdown (Figure 3C).

### Singular v dual knockdowns of SNF2L/SNF2LT and similar effects on DNA damage

**Figure 4:**
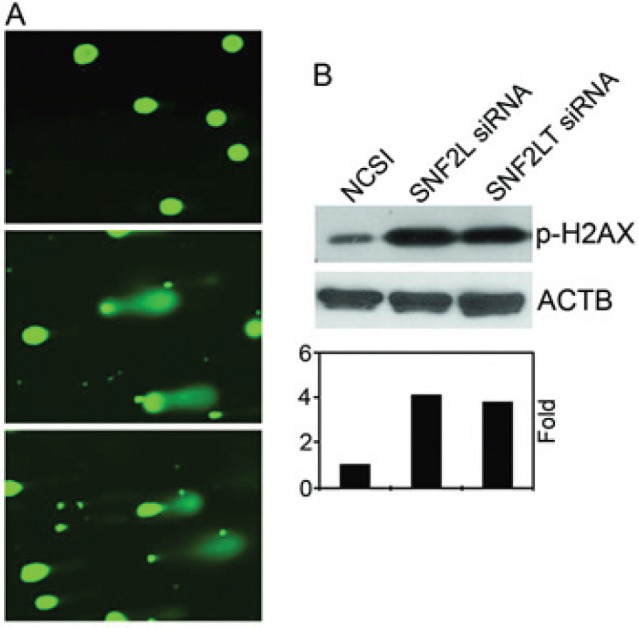
Singular v dual knockdown of SNF2L and SNF2LT and DNA damage A, MDA-MB-468 cells were transfected with SNF2L siRNA, SNF2L siRNA or NCSI. 48 hours after transfection, DNA damage was analyzed by the Comet assay and the results showed damaged DNA (the comet tail) outside the nucleus after treatment of SNF2LT siRNA (lower panel), SNF2L siRNA (middle panel) compared to undamaged DNA in the cells treated with NCSI (upper panel). B, the surrogate DNA damage gene, p-H2AX showed increased expression following either SNF2L or SNF2LT knockdown (upper panel) and increased fold expression of p-H2AX (lower panel). Each experiment was performed in triplicate and repeated at least four times.

In a recent study [21], we found that HM cell lines, although expressing SNF2L at similar levels as their normal counterparts, were exquisitely sensitive to its knockdown. Only HM lines showed significant growth inhibition, DNA damage, a DNA damage response, and phosphorylation of checkpoint proteins and marked apoptosis. In that study we believed that SNF2L knockdown triggered DNA damage which then resulted in a DNA damage response which caused a cell cycle growth arrest and the induction of apoptosis. In the present study, we wanted to compare the effects of singular knockdown of SNF2LT with singular knockdown of SNF2L on DNA damage. We also wanted to compare the effects of dual knockdowns with singular knockdowns. Singular knockdown of SNF2LT similarly triggered DNA damage as did singular knockdown of SNF2L as measured by the Comet assay (Figure 4A). This was observed in all HM lines examined. H2AX is a surrogate marker of DNA damage. DNA damage results in an immediate phosphorylation of the histone H2A family member H2AX at Ser139. Ser139-phosphorylated H2AX localizes to sites of DNA damage at subnuclear foci. We examined the level of phosphorylated H2AX (p-H2AX) by Western blotting and found that p-H2AX was significantly increased with the singular knockdowns of either SNF2L or SNF2LT in all HM lines examined (Figure 4B). Dual knockdowns of both SNF2L and SNF2LT similarly led to DNA damage determined by both the Comet assay as well as by an increase in p-H2AX (data not shown).

### Singular v dual knockdowns of SNF2L/SNF2LT and opposite effects on the DNA damage response and the cell cycle

DNA damage is thought to activate a DNA damage response, in which the center is the ATM/ATR kinase signaling pathway. ATM/ATR kinases phosphorylate the downstream effectors such as p53, Chk1, Chk2 and BRCA1. To this end, we investigated whether the critical DNA damage response network is activated by DNA damage when the DNA damage is triggered by singular v dual knockdowns of SNF2L and SNF2LT. We examined this DNA damage response in a number of different HM lines including MDA-MB-468, MDA-MB-231 and HeLa. We used western blotting to examine the levels of phosphorylated proteins of ATM, ATR, BRACA1, Chk1 and Chk2. Neither singular nor dual knockdowns of SNF2L and SNF2LT resulted in an increase in phosphorylated ATM. Singular knockdowns of SNF2L and SNF2LT however resulted in increased phosphorylations of ATR, CHK1, CHK2 but no alterations of their respective total proteins (data not shown). Interestingly while the singular knockdown of SNF2L led to an increase in phosphorylated BRCA1 [21], the singular knockdown of SNF2LT did not do so. However dual knockdowns of both SNF2L and SNF2LT did not result in increased phosphorylations of ATR, BRCA1, CHK1, CHK2 (data not shown). Therefore dual knockdowns did not result in a typical DNA damage response.

**Figure 5:**
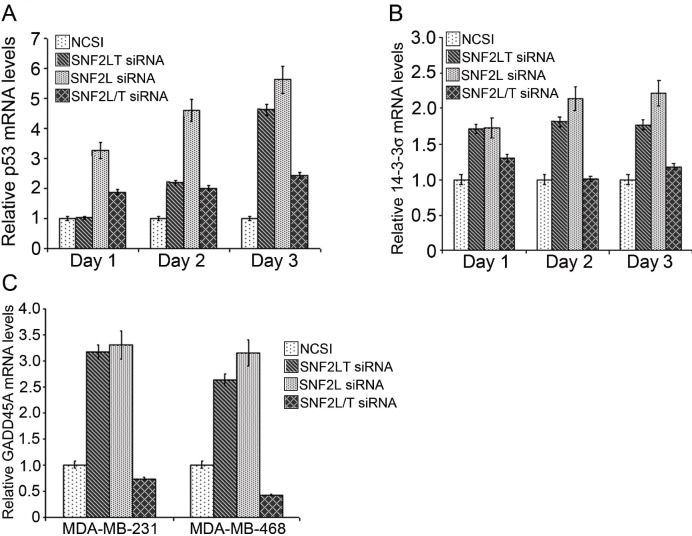
Singular v dual knockdown of SNF2L and SNF2LT and the cell cycle MDA-MB-468 cells were transfected with the different siRNAs. A, singular knockdowns of either SNF2L or SNF2LT both led to substantial increases in p53 mRNA but dual knockdowns affected p53 mRNA less so by real time RT-PCR. B, singular knockdowns of either SNF2L or SNF2LT both led to substantial increases in the p53 target gene, 14-3-3σ but dual knockdowns did not affect 14-3-3σ. C, singular knockdowns of either SNF2L or SNF2LT both led to substantial increases in another p53 target gene, GADD45A but dual knockdowns did not affect GADD45A. Each experiment was performed in triplicate and repeated at least four times.

Singular v dual knockdowns of SNF2L and SNF2LT had different effects on the triggering of cell cycle checkpoints. Singular knockdowns of SNF2L and SNF2LT resulted in nearly identical increases in relative levels of p53 mRNA (Figure 5A), 14-3-3 σ (Figure 5B) and GADD45A (Figure 5C). However dual knockdowns of SNF2L and SNF2LT resulted in no appreciable alterations in these critical cell cycle checkpoint proteins (Figure 5A; Figure 5B; Figure 5C). Singular knockdowns of SNF2L and SNF2LT also resulted in an increase in phosphorylation of p53 but dual knockdowns did not alter phosphorylation of p53 (data not shown). Since the cellular responses to DNA damage, such as cell cycle arrest, DNA repair, chromatin remodeling, and apoptosis are all thought to be well coordinated, we investigated whether singular v dual knockdowns of SNF2L and SNF2LT led to alterations in the cell cycle. Our results indicated that the HM lines examined exhibited a mild G2/M arrest with either singular SNF2L or SNF2LT knockdown. Dual knockdown did not result in a cell cycle arrest. In fact dual knockdowns resulted in an increase in proliferation (Figure 3C). Our results also showed that with singular knockdowns, the level of phosphorylated cdc2 was increased but not the level of phosphorylated Rb (Ser795) (data not shown). Since the cdc2/cyclin B kinase is thought to be pivotal in regulating the G2/M transition and Rb thought to control progression through late G1/S, our findings indicated that the effects of either SNF2L or SNF2LT inhibition on the cell cycle were largely directed at checkpoints that regulate G2/M, a finding confirmed by the mild G2/M arrest noted on flow cytometric analysis. Our observations regarding the critical targets of p53: GADD45 and 14-3-3σ also being increased in either singular SNF2L or SNF2LT knockdown but not dual knockdown again emphasize the fundamental differences between singular v dual knockdown with regard to effects on the cell cycle. Since GADD45A and 14-3-3σ both target the cyclin B/ cdc2 complex, this would appear as further confirmatory evidence that G2/M is targeted with either singular knockdown but not dual knockdown.

### Singular v dual knockdowns of SNF2L/ SNF2LT and opposite effects on apoptosis

**Figure 6:**
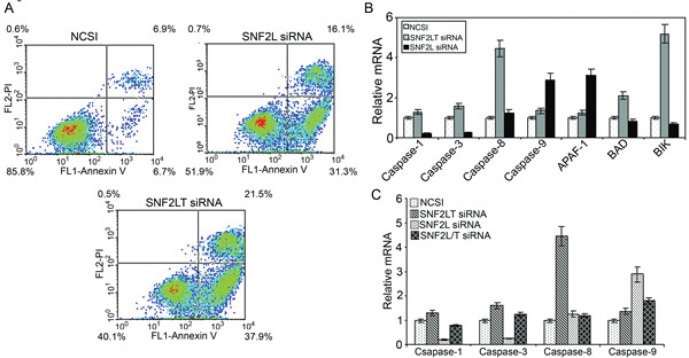
Singular v dual knockdown of SNF2L and SNF2LT and apoptosis MDA-MB-468 cells were transfected with the different siRNAs. A, MDA-MB-468 cells were monitored over the next 48-72 hours with Annexin V-FITC and propidium iodide (PI) staining. The percentage of total apoptotic cells (early and late apoptosis) is depicted. Singular knockdowns of either SNF2L or SNF2LT both led to substantial increases in both early as well as late apoptosis. Dual knockdowns did not result in apoptosis over levels induced by the NCSI negative control (data not shown). During this time period the relative mRNA levels of potential genes involved in the apoptotic process was surveyed. B, singular knockdown of SNF2L led to a significant increase in Apaf-1 and caspase 9 whereas singular knockdown of SNF2LT led to a significant increase in caspase 8, BAD and BIK. C, dual knockdowns compared to NCSI knockdown did not result in a significant increase in any caspase. Each experiment was performed in triplicate and repeated at least four times.

Staining with FITC-conjugated annexin V and propidium iodide (PI) was used to identify subpopulations of cells with apoptosis. With singular knockdowns of either SNF2L and SNF2LT using MDA-MB-468 cells, a significant and near equivalent degree of apoptosis was induced (Figure 6A). Dual knockdowns did not result in apoptosis over levels induced by the NCSI negative control. We applied real-time PCR to detect which apoptotic pathway was activated when either SNF2L or SNF2LT was knocked down. Although either singular knockdown resulted in the near equivalence in the degree of apoptosis, different apoptotic pathways appeared involved. With SNF2L knockdown (Figure 6B), increased Apaf-1 and increased caspase-9 expression was observed. With SNF2LT knockdown, we found instead that caspase 8, BAD and BIK increased (Figure 6B). Therefore, the apoptotic pathway triggered by SNF2LT knockdown appeared different from the apoptotic pathway caused by SNF2L knockdown. Dual knockdowns did not appreciably activate any caspase pathway and were nearly identical to NCSI knockdown (Figure 6C). Schematic summarizes our findings (See Supplementary Information on line).

### SNF2LT overexpression and apoptosis

Based on our prior experiments we reasoned that since SNF2L knockdown inhibited cell growth, that SNF2L overexpression might be expected to enhance cell growth. To investigate whether the expression of SNF2LT would promote cell growth, we constructed an expression vector that overexpressed SNF2LT both constitutively and conditionally. We examined the effects of SNF2LT overexpression by both transient as well as stable transfections. Surprisingly we did not observe an increase in cell growth but rather an inhibition of cell growth and an induction of apoptosis. From this we reasoned that it was the ratio of SNF2LT to SNF2L that determined cell proliferation v apoptosis and that the singular overexpression of either full length SNF2L or its truncated isoform, SNF2LT was the ratio equivalence of singular knockdowns with the result being cell cycle arrest and cell death (See Supplementary Information on line).

## DISCUSSION

Epigenetic changes in gene expression play important roles in the development, progression and ultimate therapeutic targeting of human cancers [29-31]. In addition to the major mechanisms of DNA methylation and histone modification thought to regulate epigenetic changes [2,3,32,33], altered nucleosome positioning through chromatin-remodeling complexes are playing increasingly prominent roles in this area [4,5,11,12,13]. Loss of SNF2L complex activity could represent a novel mechanism for altering gene expression during tumor progression. Similarly other types of SNF2L complex alterations could prove deleterious to cancer cells. The SNF2L complex itself could therefore be a potential therapeutic target.

In studying SNF2L, we discovered a novel truncated isoform, SNF2LT which formed the basis of the present study. When compared to full length SNF2L, SNF2LT lacked three important domains: HAND, SANT and SLIDE. Truncated isoforms usually have antagonistic effects, eg., dominant negative effects, on their full length molecule. Here SNF2LT seemed synergistic. However, we compared the effects of SNF2LT knockdown with the effects of SNF2L knockdown and although there were some minor differences in the changes effected by SNF2L v SNFLT knockdown on select cell cycle proteins, eg. p-BRCA1 and apoptosis pathways triggered, eg. caspase 9 v caspase 8, there was much more in common between singular SNF2L v singular SNF2LT knockdown in inducing DNA damage, a DNA damage response, cell cycle arrest and apoptosis selectively in cancer cell lines. Therefore SNF2LT’s effects on SNF2L certainly were not of a dominant negative nature. The effects of dual knockdowns of SNF2L and SNF2LT were very different than their singular knockdowns. Dual knockdown induced DNA damage but did not result in a DNA damage response, a cell cycle arrest or apoptosis. In fact cancer cell lines subjected to dual knockdown paradoxically exhibited increased cell growth. Our findings indicated that SNF2L and its isoform tightly regulate the cancer cell’s response to DNA damage. Cancer cell lines which endogenously express low levels of both SNF2L and its isoform would mimic the situation of dual knockdown and would experience DNA damage allowed to propagate unchecked.

SNF2LT is not the only alternatively spliced isoform of SNF2L to have been described but it is perhaps the most important isoform because of its near ubiquity of expression, the similar functional consequences of its singular knockdown compared with SNF2L knockdown and its presumed interactions (direct or indirect) with the full length molecule. A number of other alternatively spliced variants of SNF2L expressed in multiple cell types and exhibiting different subcellular localizations and functions have been described [34,35]. These specific isoforms were generated through the alternate use of exons 1 and 13, and by the use of alternate donor splice sites within exon 24. Alternate splicing within exon 24 removed a NLS sequence and altered the subcellular distribution of the SNF2L protein [34,35]. Still another splice variant of human SNF2L called SNF2L +13 which contained a nonconserved in-frame exon within the conserved catalytic core domain of SNF2L has been described [22]. This latter variant of SNF2L, SNF2L + 13, retained its ability to incorporate into multiprotein complexes but was devoid of enzymatic activity. This SNF2L + 13 splice variant was predominately found in nonneuronal cells of the nervous system. None of these other splice variants exhibited the near ubiquity of expression of SNF2LT. None of these other splice variants have been demonstrated to have effects on DNA damage, the DNA damage response and the cell cycle. None of these other splice variants have been shown to interact directly or indirectly with full length SNF2L.

Both the relative as well as the absolute amounts of SNF2L and its isoform SNF2LT are obviously critical to their functions. When the relative amounts (their ratios) were altered through knockdown or overexpression, HM lines responded by DNA damage, a DNA damage response, cell cycle arrest and apoptosis. When the absolute amounts were altered through either dual knockdown or, presumably, in the natural situation of endogenously low levels of expression of both SNF2L as well as SNF2LT, such as occurs in MARY-X and its derived spheroids, the response would be different: DNA damage but no DNA damage response, no cell cycle arrest and no apoptosis. In MARY-X lymphovascular emboli and its *in vitro* derived spheroids thought to be equivalent to each other [36], DNA damage would be allowed to propagate unchecked.

The singular effects of SNF2LT and SNF2L knockdown on DNA damage, the DNA damage response, the cell cycle and apoptosis, while strikingly similar, did exhibit some differences. For example, while the singular knockdown of SNF2L led to an increase in p-BRCA1 [21], the singular knockdown of SNF2LT did not do so. The latter results suggested that the knockdown of SNF2LT might selectively block the DNA repair pathway involving p-BRCA1. This could explain why the growth of the cells subjected to SNF2LT knockdown were even more reduced than the growth of the same cells subjected to SNF2L knockdown. Another example of differences between SNF2L and SNF2LT knockdown was not in the triggering of apoptosis but rather in the pathway of apoptosis which was triggered [37-38]. With SNF2L knockdown, Apaf-1 was activated which, in turn, activated caspase-9 and the rest of the caspase cascade including caspase-3. With SNF2LT knockdown, caspase-9 was not activated. Instead, caspase-8, BAD and BIK were activated. BAD is a proapoptotic member of the Bcl-2 family that promotes cell death by displacing BAX from binding to Bcl-2 and Bcl-xL. BIK/Nbk (Bcl-2-interacting killer/natural born killer) is a potent pro-apoptotic protein belonging to a group of the Bcl-2 family. Functionally, BIK is able to bind to and antagonize anti-apoptotic Bcl-2 family members including Bcl-2 and Bcl-xL. The apoptotic pathway triggered by SNF2LT knockdown therefore differed from that triggered by SNF2L knockdown.

Despite the differences between SNF2LT and SNF2L knockdown with respect to certain aspects of the DNA damage response, ie., p-BRCA1 and the differing pathways of apoptosis, singular SNF2LT and SNF2L knockdowns had much more in common and this common response involved alterations in both the levels of p53 as well as its degree of phosphorylation. This common p53 response to either SNF2LT or SNF2L knockdown, in turn, suggests two possible mechanisms:

### 

#### Mechanism #1

The inhibition of expression of SNF2LT or SNF2L leads to functional losses of SNF2LT or SNF2L or the complexes containing them which then directly causes DNA damage, which, in turn, activates the DNA damage response. In this DNA damage response, p53 is activated through phosphorylation on Ser15 by ATM/ATR and on Ser20 by Chk1/Chk2. p53 plays a very important role in responding to DNA damage and promoting/maintaining checkpoint arrest [39]. For example, phosphorylated p53 activates its key transcriptional targets, GADD45A and 14-3-3σ [40]. GADD45A causes the dissociation of the Cdc2 and cyclin complex and 14-3-3σ sequesters the cyclinB/Cdc2 complex in the cytoplasm.

#### Mechanism #2

The inhibition of expression of SNF2LT or SNF2L directly activates the expression of p53. Either mechanism may be occurring singly or in combination with either SNF2LT or SNF2L knockdown.

SNF2LT is a novel alternatively spliced truncated isoform of human SNF2L that lacks the three C-terminal structural domains: HAND, SANT and SLIDE. These three domains are tightly connected and move as one unit during the remodeling process. SANT domains of other proteins, in particular, have been shown to bind histone tails and the histone H4 tail is important for ISWI-driven nucleosome remodeling [41]. Deletion of the H4 tail or grafting the tail onto another histone abolishes ISWI ATPase stimulation and nucleosome sliding [18]. This means that SNF2LT loses some very important functions: binding to and moving along DNA during the remodeling process and binding to histone, in which the binding may be important for nucleosome remodeling. And yet, SNF2LT knockdown is the near equivalent of SNF2L knockdown. How can these observations be reconciled? Obviously it is important to understand all of the interactions between SNF2L and its truncated isoform, SNF2LT in order to reconcile these observations. SNF2L and SNF2LT may bind each other and form a complex with BPTF and RbAp46/RbAp48. In this complex, SNF2LT may modulate the function of SNF2L and vice versa, adding an additional layer of fine-tuned specificity in ATP-dependent chromatin remodeling. Certainly the similarities in DNA damage, the DNA damage response, cell cycle arrest and apoptosis with either type of singular knockdown suggest that SNF2L does not directly interact with SNF2LT in a dominant negative manner. But SNF2LT may directly interact with SNF2L in a different manner in forming the complex with BPTF and RbAp46/RbAp48 mentioned above. Co-immunoprecipitation experiments of endogenous substrates which bind SNF2L and/or its isoform would further support or refute such direct interactions.

The disparate effects of SNF2LT/SNF2L dual v singular knockdowns, on the other hand, raise the distinct possibility of a type of indirect interaction between SNF2LT and SNF2L. To further support this type of indirect interaction, one approach would be to analyze expression profiles following SNF2L knockdown, SNF2LT knockdown and dual knockdown determining their degree of overlap. Experiments are presently in progress to determine whether the interactions of SNF2L with its truncated isoform, SNF2LT are direct or indirect or both.

The existence of a functional splice variant of SNF2L, SNF2LT that acts in cohort with SNF2L suggests an additional level of complexity possibly related to their biology. There are many examples in nature where master orchestration of diverse biological functions such as immune homeostasis, innate immunity and global gene expression involve regulation by splice isoform variants. Such examples include FOXP3 and exon 2 deleted FOXP3Δ2 [42], the toll-like receptor (TLR) and its alternatively spliced variants [43] and, in this case, SNF2L and its truncated isoform, SNF2LT. In all these examples, it seems as if the greater the master orchestration, the greater is the level of regulatory complexity.

## Supplementary Figures and Section




