# Supplementary material for: Singular v Dual inhibition of SNF2L and its isoform, SNF2LT, have similar effects on DNA Damage but opposite effects on the DNA Damage Response, Cancer Cell Growth Arrest and Apoptosis
Source: Oncotarget. 2012 May 9;3(4):475–89. doi: 10.18632/oncotarget.479 (PMC3380581; doi:10.18632/oncotarget.479)
Supplement: Supplementary file 1 [file oncotarget-03-475-s001.pdf]

Singular v Dual inhibition of SNF2L and its isoform, SNF2LT, have similar effects on DNA Damage but opposite effects on the DNA Damage Response, Cancer Cell Growth Arrest and Apoptosis – Ye et al

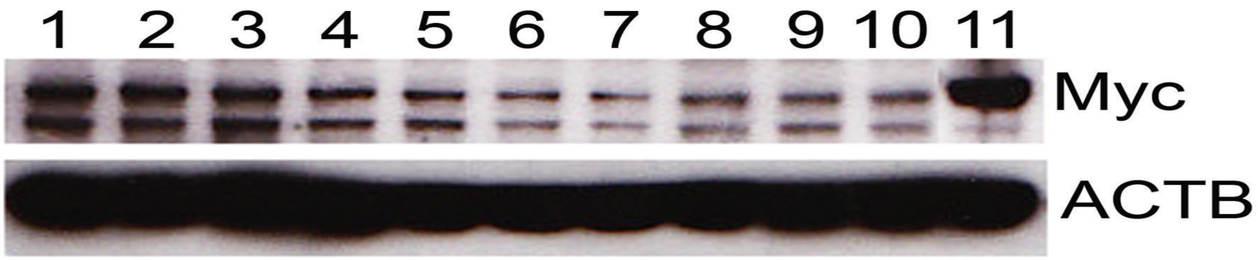

Supplementary Figure 1

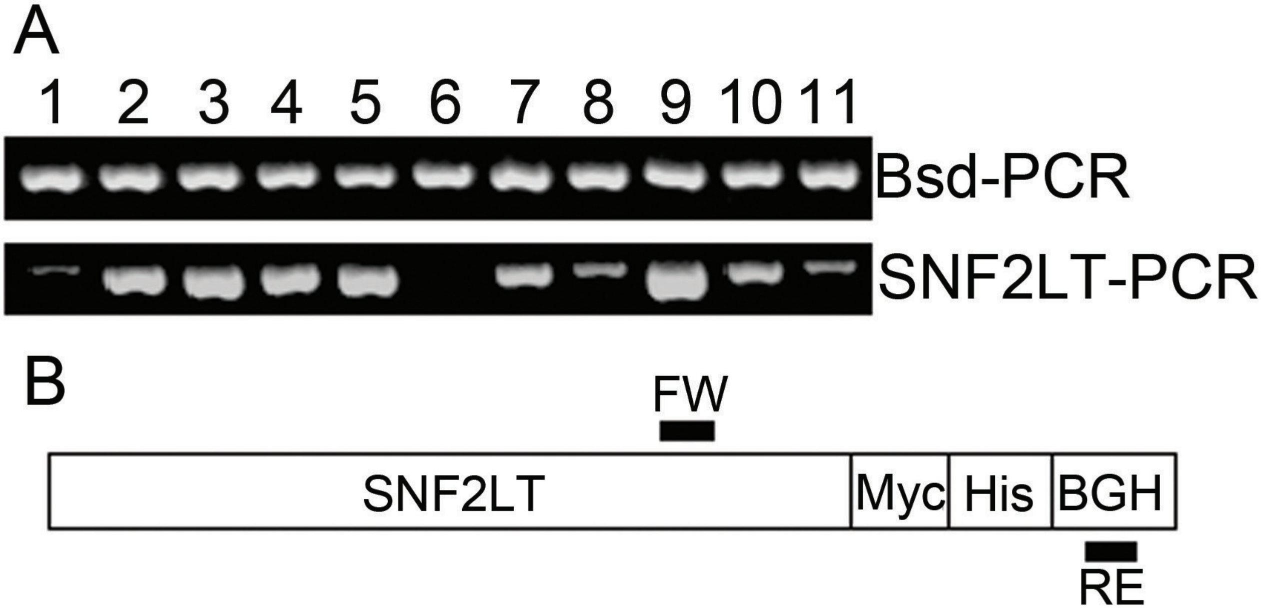

Supplementary Figure 2

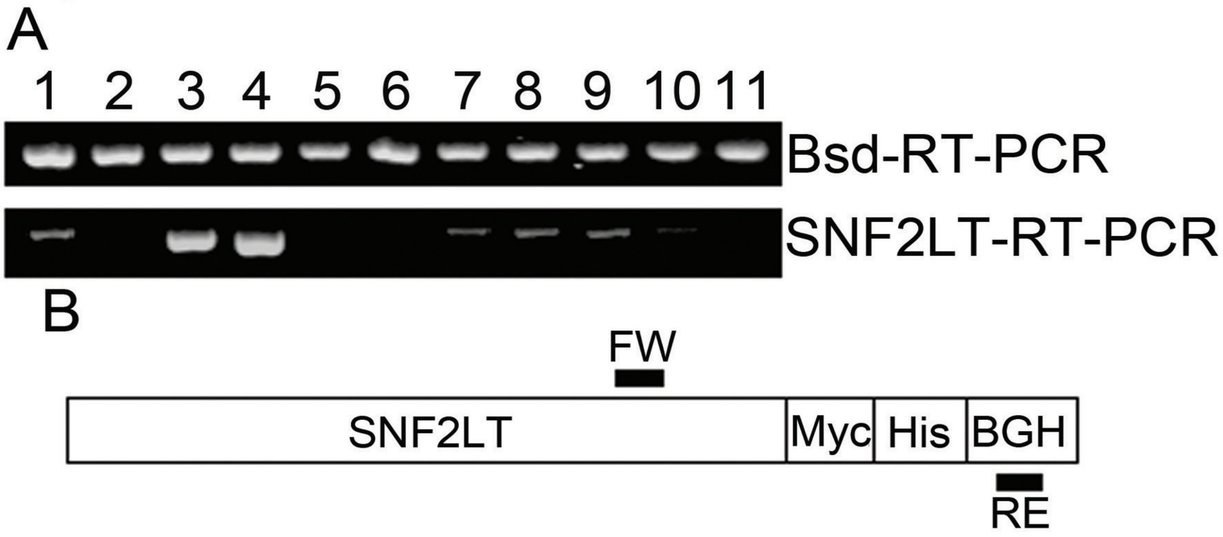

Supplementary Figure 3

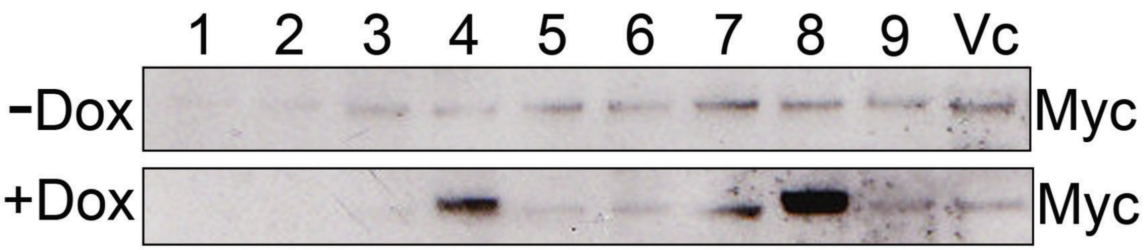

Supplementary Figure 4

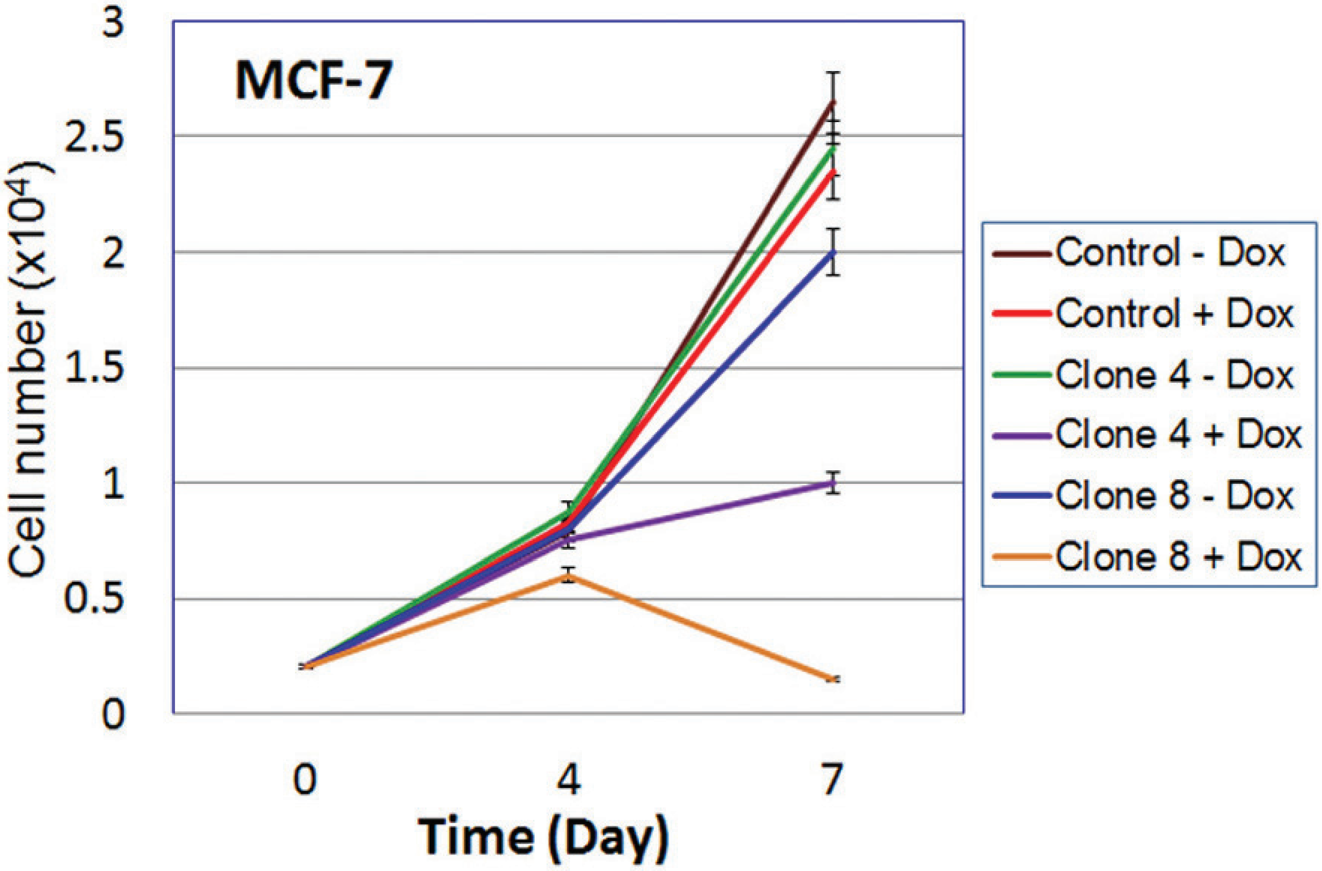

Supplementary Figure 5

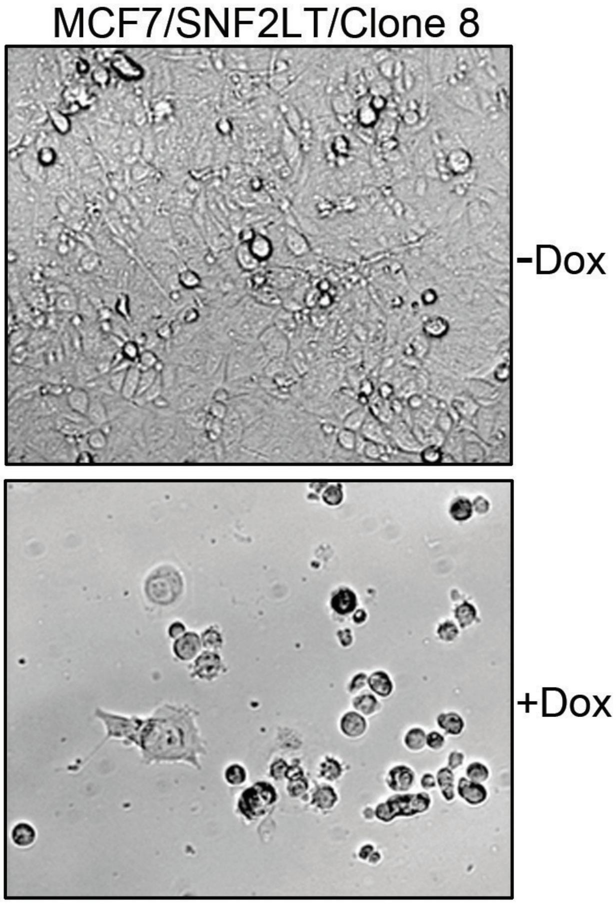

Supplementary Figure 6

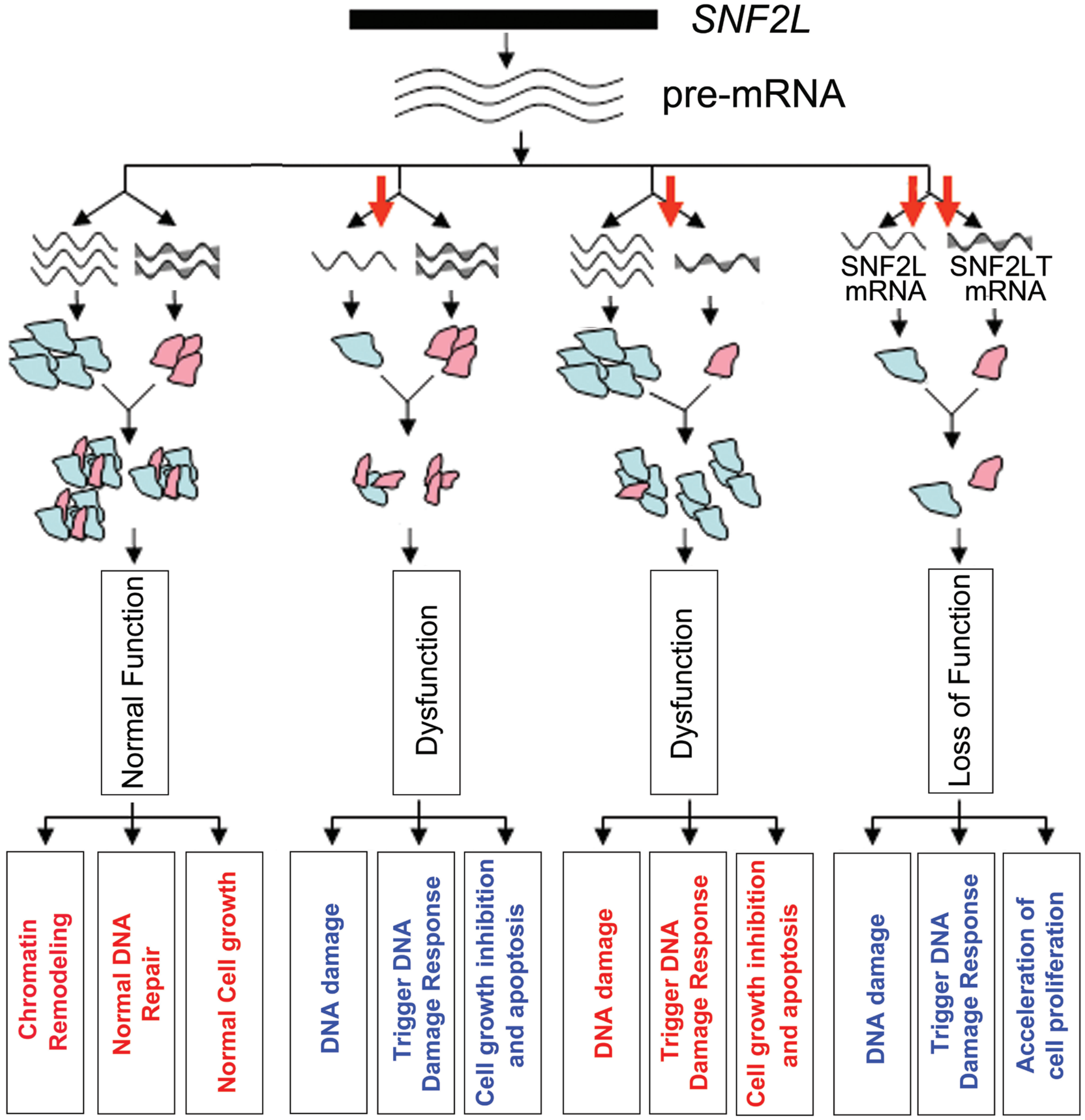

Supplementary Figure 7
