# Supplementary material for: Singular v Dual inhibition of SNF2L and its isoform, SNF2LT, have similar effects on DNA Damage but opposite effects on the DNA Damage Response, Cancer Cell Growth Arrest and Apoptosis
Source: Oncotarget. 2012 May 9;3(4):475–89. doi: 10.18632/oncotarget.479 (PMC3380581; doi:10.18632/oncotarget.479)
Supplement: Supplementary file 2 [file oncotarget-03-475-s002.pdf]

Singular v Dual inhibition of SNF2L and its isoform, SNF2LT, have similar effects on DNA Damage but opposite effects on the DNA Damage Response, Cancer Cell Growth Arrest and Apoptosis – Ye et al

SUPPLEMENTARY INFORMATION

Studies on the constitutive overexpression of SNF2LT

Our aforementioned results had indicated that inhibition of either SNF2L or SNF2LT led to suppression of cell growth and induction of apoptosis but that inhibition of both SNF2L and its truncated isoform did not. Since the singular knockdowns of either SNF2L or its isoform, SNF2LT, in effect, change the ratio of SNF2L to its isoform, we decided to examine this ratio in a different way. Previously we had overexpressed SNF2L through both transient and stable transfections and observed the induction of apoptosis (21). In the present study we examined the effects of SNF2LT overexpression by both transient as well as stable transfections into both the MCF-7 and the MDA-MB-468 lines. We chose both of these cell lines because they exhibited a variation in the endogenous levels of SNF2LT expression ranging from little to no expression in MCF-7 and reasonably high expression in the MDA-MB-468. Full-length SNF2LT ORF was cloned into pcDNA6/Myc-His-A (Invitrogen, Inc., Carlsbad, CA) to construct expression vector pcDNA6/SNF2LT-Myc-His, in which SNF2LT was fused in frame with the myc epitope and the C-terminal polyhistidine tag (His). Here, we used flags fused with SNF2LT because we did not have an antibody specifically against SNF2LT. The fusion gene SNF2LT-Myc-His was confirmed by sequencing. We first performed transient transfection experiments. In transient expression using the MDA-MB-468 line, the overexpressed product of SNF2LT-Myc-His was detected by Western Blot using an antibody against Myc (Invitrogen) (Supplementary Figure 1). The results with the MCF-7 line were similar. To test the effect of SNF2LT overexpression on DNA stability and cell growth, we screened for single stable clones that overexpressed SNF2LT. The vector pcDNA6/SNF2LT-Myc-His was transfected into MDA-MB-468 and Blasticidin was applied to screen for positive clones. Greater than 80 blasticidin-resistant clones emerged and were tested by Western Blot. Not one was found to express SNF2LT-Myc-His fusion protein (data not shown). The results with MCF-7 cells were similar. Considering that Blasticidin is a perfect antibiotic marker to screen for positive clones, we doubted that all the tested clones (more than 80) would be falsely Blasticidin resistant. We believed that these seemingly Blasticidin resistant clones were indeed Blasticidin resistant where the Blasticidin gene was properly expressed but that SNF2LT was not expressed. To test our hypothesis, we isolated the genomic DNA and total RNA from these Blasticidin-resistant clones. We first used PCR to detect the insertion and the integration of the expression vector into the genomic DNA. The results indicated that the vast majority of clones were positive for the integration of the Blasticidin resistance gene (Bsd) and SNF2LT-Myc-His fusion gene (Supplementary Figure 2A). RT-PCR was then used to detect the transcription of Bsd and SNF2LT-Myc-His fusion genes. We found that level of Bsd mRNA was high but the level of SNF2LT-Myc-His mRNA was low in most clones and absent in some (Supplementary Figure 3A). In the PCR (Supplementary Figure 2B) or RT-PCR (Supplementary Figure 3B) analysis of SNF2L-Myc-His DNA or cDNA, the forward primer was located in the SNF2LT ORF and the reverse primer in the BGH region of vector, in order to be able to amplify the template from only the successfully transfected or expressed SNF2LT-Myc-His fusion gene. The results indicated that, in most Blasticidin-resistant clones, both the Bsd gene and the SNF2LT-Myc-His fusion gene were indeed inserted and integrated into the cellular genome (Supplementary Figure 2A) and that the Bsd gene was indeed expressed (Supplementary Figure 3A). However none of the clones produced any SNF2LT-Myc-His fusion protein (data not shown), though most harbored the integrated SNF2LT-Myc-His fusion gene (Supplementary Figure 2A) and some even transcribed it (Supplementary Figure 3A). Based on these results and our previous results that we had confirmed the right sequence of the SNF2LT-Myc-His fusion gene before transfection, and that we had detected SNF2L-Mys-His fusion protein in the transient transfection experiments, we ruled out the possibility that the failure to express the SNF2LT-Myc-His fusion protein was from defective sequence, vector, transfection or screening. We believe that our findings support the conclusion that SNF2LT overexpression is “toxic” to the cells, that SNF2LT overexpression alters the ratio of SNF2LT to SNF2L and that this leads to the instability of genomic DNA, DNA damage, a DNA damage response and cell cycle arrest. The clones overexpressing SNF2LT eventually went on to apoptosis during the prolonged time interval of Blasticidin selection and only those clones not overexpressing SNF2LT protein and therefore those clones with an unaltered ratio of SNF2LT to SNF2L would survive.

Studies on the conditional overexpression of SNF2LT

From the aforementioned experiments where constitutive gene expression was utilized, we did not obtain clones that stably expressed SNF2LT and therefore could not directly examine the effects of the overexpression of SNF2LT on cell growth. So we then tried to use a conditional expression system to study the effects of SNF2LT overexpression. The Tet-On inducible gene expression system was chosen for this latter study. Considering that Blasticidin is a good selective marker, we constructed a new Tet-on inducible gene expression vector, pP_{Tight}-Myc-His-A, by using inducible promoter P_{Tight} consisting of a modified Tet-Responsive Element (TRE) from the vector pmRi-mCherry (Clontech, Inc., Mountain View, CA) to replace the constitutive promoter P_{CMV} of pcDNA6/Myc-His-A (Invitrogen). The mCherry gene from vector pmRi-mCherry was then subcloned into pP_{Tight}-Myc-His-A to form inducible reporter gene expression vector pP_{Tight}-Myc-His-mCherry. We used this inducible reporter vector to test if the gene expression through this vector was inducible in the presence of DOX. This reporter vector was transfected into the MCF-7 Tet-on Advanced Cell Line (Clontech, Inc.) and DOX (1 µg/ml) was added to the cells. The reporter gene mCherry was virtually silent (no red fluorescence) in the absence of DOX, but obtained high expression in the presence of DOX (data not shown), indicating that this vector was suitable for studying inducible gene expression. We then subcloned the SNF2LT gene into pP_{Tight}-Myc-His-A to form an inducible SNF2LT expression vector, pP_{Tight}-SNF2LT-Myc-His, in which SNF2LT ORF was fused in frame with tags Myc and His. The vector pP_{Tight}-SNF2LT-Myc-His was transfected into MCF-7 Tet-on cells (Clontech, Inc.), and Blasticidin was applied to screen for the positive clones. In the experiment, the blank vector pP_{Tight}-Myc-His-A was used as a negative control. 30 Blasticidin-resistant clones were selected for analysis of SNF2LT expression using Western Blot with antibody against Myc (Invitrogen, Inc.). Our results showed that several clones expressed SNF2L-Myc-His fusion protein in the presence of DOX (Supplementary Figure 4). Among these clones, Clones 4 and 8 had the highest SNF2LT-Myc-His fusion protein expression. We then selected Clones 4 and 8 to examine the effect of SNF2LT overexpression on cell growth. Our results showed that cell growth was inhibited when SNF2LT was overexpressed in the presence of DOX (Supplementary Figure 5). In the cells transfected with blank vector pP_{Tight}-Myc-His, there was no difference in cell growth with or without treatment by DOX. However, in Clones 4 and 8, the cell growth was much slower in the group treated by DOX, indicating that induced SNF2LT overexpression had indeed severe inhibitory effects on cell growth. In Clone 8 in which SNF2LT expression was highest with DOX induction, not only was there growth inhibition but the cell numbers were reduced by day 7 below starting numbers, indicating induction of cell death (apoptosis). Using phase contrast microscopy, increased cell death was observed with DOX induction (Supplementary Figure 6). It is our belief that both constitutive and conditional overexpression of SNF2LT again changes the ratio of SNF2LT to SNF2L giving us the equivalence of singular knockdown with the result being cell cycle arrest and cell death.

Schematic of the hypothesis

Schematic (Supplementary Figure 7) depicts the differences among no knockdowns, singular knockdowns of either full length SNF2L or its SNF2LT isoform and dual knockdowns on DNA damage, the DNA damage response, growth inhibition and apoptosis. Although the exact mechanisms responsible for these disparate responses remain to be elucidated, acknowledgement of both the existence of the truncated isoform as well as the different effects of its singular *v* dual knockdown begin to provide insights into these mechanisms.
